# Mining the Protein Data Bank to inspire fragment library design

**DOI:** 10.3389/fchem.2023.1089714

**Published:** 2023-02-10

**Authors:** Julia Revillo Imbernon, Luca Chiesa, Esther Kellenberger

**Affiliations:** Laboratoire d’Innovation Thérapeutique, Faculté de Pharmacie, UMR7200 CNRS Université de Strasbourg, Illkirch-Graffenstaden, France

**Keywords:** scaffold, binding mode, site comparison, interaction graph, similarity, FBDD, PDB

## Abstract

The fragment approach has emerged as a method of choice for drug design, as it allows difficult therapeutic targets to be addressed. Success lies in the choice of the screened chemical library and the biophysical screening method, and also in the quality of the selected fragment and structural information used to develop a drug-like ligand. It has recently been proposed that promiscuous compounds, i.e., those that bind to several proteins, present an advantage for the fragment approach because they are likely to give frequent hits in screening. In this study, we searched the Protein Data Bank for fragments with multiple binding modes and targeting different sites. We identified 203 fragments represented by 90 scaffolds, some of which are not or hardly present in commercial fragment libraries. By contrast to other available fragment libraries, the studied set is enriched in fragments with a marked three-dimensional character (download at 10.5281/zenodo.7554649).

## 1 Introduction

Over the past 25 years, the fragment-based approach has demonstrated its value to drug discovery by producing fifty-eight successful fragment-to-lead developments, including six approved drugs (Erlanson, 2021; de Esch et al., 2022). A fragment-based drug discovery (FBDD) project begins with the discovery of a molecule characterized by low molecular weight, low chemical structure complexity, and low binding affinity for the assayed protein target (Keserü et al., 2016; Li, 2020). It generally relies on biophysical methods, which are sensitive, and three-dimensional binding mode information, which facilitates the elaboration of the fragment hit into a drug-like lead. Compared to high-throughput screening campaigns, fragment screening has the advantage of a reduced scale for screening while efficiently exploring chemical space, the possible combinations between fragments giving access to multiple complex compounds (Shi and von Itzstein, 2019).

The fragment quality is critical to the success of drug design. Fragment must be stable in buffers and biological media, have high solubility in water, and have a well-defined synthetic pathway to allow for follow-up discovery to develop a drug candidate. Accordingly, fragment libraries present a preferred range of physico-chemical descriptors, e.g., following the rule of three (molecular weight ≤300; number of hydrogen bond donors ≤3; number of hydrogen bond acceptors ≤3; logP ≤3; number of rotatable bonds ≤3), and have generally been filtered to exclude unwanted compounds, e.g., reactive or poorly soluble compounds (Congreve et al., 2003). Recommended cut-off values for molecular weight have fluctuated in response to questions about ideal size and complexity.

In 2021, approximately half a million fragments were marketed in 81 libraries by 14 chemical suppliers (Revillo Imbernon et al., 2022). Collections vary both in size and content. Large catalogs, which contain up to 172,000 fragments, allow the assembly of custom libraries based on specific criteria. Numerous medium to small size libraries (80–8,968 fragments) are adapted to particular purposes, e.g., collections of fluorinated fragments are suitable for screening by NMR (Troelsen and Clausen, 2020; Shinya et al., 2022). Several commercial libraries focus on diversity, aiming for good fragment space coverage and the ability to deliver hits to most targets. Academic initiatives have also proposed libraries of fragments built by maximizing structural or shape diversity, such as libraries developed based on diversity-oriented synthetic (DOS) strategy (Kidd et al., 2018). The diverse fragment libraries can serve different purposes, ranging from being a tool to study ligand-protein interactions or to facilitate bioisosteric replacement (Heidrich et al., 2019; Ferri et al., 2020) to the screening collection applicable to a wide variety of target proteins, such as the Diamond iNext Poised library of the Structural Genomics Consortium (DSiP) and the F2X chemical library, which both have been designed taking into account the diversity of chemical structures (Cox et al., 2016; Wollenhaupt et al., 2020). The diversity of chemical structures, however, does not presage the ability to provide hits for different proteins, as structurally dissimilar fragments may exhibit the same biological activity. XChem’s analysis of DSiP screening campaigns thus demonstrated that structurally dissimilar fragments can form the same interactions, consistent with the pharmacophore concept (Carbery et al., 2022). The investigation of 309 protein-fragment structures obtained for 10 unrelated targets and 225 fragments led the authors to propose a strategy for selecting functionally diverse fragments based on the interactions formed with multiple proteins, as encoded in interaction fingerprints. Applied to DSiP hits, it yielded an ensemble of hundred functionally diverse fragments. The pharmacophore concept is also the basis for the development of the SpotXplorer library, which is composed of 96 fragments selected from commercial collections in order to maximize pharmacophore diversity while covering as much as possible of the 425 2-point and 3-point binding pharmacophores present in the Protein Data Bank (PDB) hotspots (Bajusz et al., 2021). SpotXplorer library has delivered hits for both well-studied proteins (G-protein coupled receptors and kinases) and novel targets (e.g., histone methyl transferase).

In this study, we further mine the PDB for relevant fragments, looking for small molecules able to adapt their binding mode to the protein context. We thus systematically compared the binding modes as well as the binding sites of a fragment if present in more than one PDB entry. We identified 203 fragments exhibiting two or more binding modes and binding to two or more different protein sites. This set of versatile PDB fragments overlaps with commercial libraries, contains approved drugs and approved drugs substructures, and reveals scaffolds that are still little exploited.

## 2 Material and methods

### 2.1 Collection of versatile PDB fragments

Fragment is here defined as a small molecular compound (molecular weight below 300 Da, non-hydrogen atoms count ranging from 2 to 18) that is not a monosaccharide, an organometallic compound, a prosthetic group, a crystallization additive (solvent, detergent, buffer, polyalcohol, etc.) (Desaphy et al., 2015; Drwal et al., 2018), a simple polyatomic ion (phosphate, sulfate or carboxylic acid), a small polyhalogenated compound, or a linear aliphatic compound.

Three-dimensional structures of protein in complex with a fragment were selected in the PDB if the deposition date is after 1 January 2000 and the resolution is below 3 Å. Their mmCIF files were downloaded from the RCSB website (Berman et al., 2000; RCSB, 2022). The preparation and standardization of the data followed the previously published protocol for the creation and maintenance of the sc-PDB, which provides, in an all-atom and annotated description, the druggable sites of the PDB and their ligands (Kellenberger et al., 2006; Desaphy et al., 2015). Briefly, the entries were filtered on the basis of molecular completeness and hydrogen atoms were added using protoss (v4.0, ZBH, University of Hamburg, Germany) (Bietz et al., 2014). For each PDB entry, the fragments, and their protein target (here including only amino acid residues) were saved in separate MOL2 files.

The frequency of each fragment in the data sample was evaluated by considering the occurrence of its HET code. Only fragments present in more than one PDB file were further considered.

The binding mode of a fragment to a protein site was described as an interaction graph. Hydrogen bond, ionic bond, aromatic stacking, and hydrophobic contacts were detected based on geometric rules using IChem (v5.2.8, University of Strasbourg, France) (Desaphy et al., 2013; Da Silva et al., 2018). For each interaction, a pseudo-atom is placed on the ligand interacting atom and labeled according to the nature of the interaction. In the case of hydrogen bond, a second pseudo-atom is defined halfway to the protein interacting atom to account for the directionality of the bond. The pseudo-atoms constitute the nodes of the interaction graph. Edges are defined between all pairs of nodes and labeled with the corresponding Euclidian distance. All graphs generated for the same fragment were compared to each other using a Subgraph Matching Kernel (max nodes = 3). Edge similarity was obtained *via* a normalized distance function (max distance = 1) (Siglidis et al., 2020). The kernel yields a similarity score value ranging between 0 and 1. The number of binding modes of a fragment was inferred from density-based clustering using Sklearn (eps = 0.23, N = 2) (Pedregosa et al., 2011). A correction of this number was applied based on shape similarity using ROCS (v3.4.1, OpenEye, Cadence Design Systems, Inc.). Two clusters were merged if the graphs of one cluster are subgraphs of the other cluster (TverskyCombo score ≥2). Only fragments showing two or more binding modes were further considered.

The binding cavity of a fragment was described as a cloud of points colored with pharmacophoric properties using Volsite tool in IChem and considering a maximal distance of 4 Å between the points and the non-hydrogen atoms of the fragment (Desaphy et al., 2012; Da Silva et al., 2018). All clouds of points generated for the same fragment were compared to each other using ProCare (Eguida and Rognan, 2020). Procare benchmarking, conducted by its authors, indicated that the comparison of dissimilar cavities yields a score below 0.47. Comparisons were made twice (cavity 1 vs*.* cavity 2 and cavity 2 vs*.* cavity 1), and the best score was retained. The number of cavities of a fragment was inferred from density-based clustering using Sklearn (eps = 0.47, N = 2) (Pedregosa et al., 2011). Only fragments showing two or more binding cavities were further considered.

Pan-assay interference compounds (PAINS) (Baell and Holloway, 2010) were identified based on 652 rules using Filter (v.2.5.1.4; Openeye Scientific Software, Santa Fe) and removed from the dataset.

### 2.2 Analysis of versatile PDB fragments

Molecular descriptors were computed for the PDB fragments using Pipeline Pilot (v.22.1.0.2935, BIOVIA, Dassault Systèmes, Pipeline Pilot): molecular weight, AlogP, molecular polar surface area, number of hydrogen bond acceptors and donors, aqueous solubility, number of positively and negatively charged atoms, number of rotatable bonds, number of rings, number of stereocenters, number of heavy atoms and solvent accessible surface area. The Plane of Best Fit (PBF) was calculated using Openeye libraries (v2019.10.2, OpenEye, Cadence Design Systems, Inc.) from a low energy conformer generated by corina (v3.40, Molecular Networks GmbH, Nürnberg, Germany).

The modeling confidence of the binding sites and fragments was assessed by the Real Space Correlation Coefficient (RSCC). The validation XML files of each PDB entry was downloaded from the PDB website (RCSB, 2022), and the mean RSCC of the residues in the fragment binding site was calculated. The coefficient of the fragment was directly extracted from the validation XML file. Model’s quality was assigned based on Twilight classification: RSCC >0.9 for a model that fits the density, 0.9 ≥ RSCC ≥0.8 for a model that partially fits the electronic density and RSCC <0.8 for a model with significant parts are missing from the density (Weichenberger et al., 2013; Deller and Rupp, 2015).

The sequence conservation between the proteins binding the same fragment was evaluated by local alignment using the EMBOSS water package (v.6.6.0.0, EMBL-EBI, Cambridgeshire, United Kingdom). EBLOSUM62 was used as score matrix. Penalties for gap opening and gap extension were set to 10 and 0.5, respectively. For each PDB complex of a fragment, the protein chain containing more than half of the amino-acids forming the fragment binding site was saved in FASTA format. Proteins were clustered based on sequence identity through density-based clustering using Sklearn (eps = 0.10 or eps = 0.75, N = 2) (Pedregosa et al., 2011). Only alignments which contains at least hundred amino-acids were considered.

The structural conservation between the proteins binding the same fragment was evaluated by local 3D-alignment using CE with default settings (v1.02.2, San Diego Supercomputer Center, United States). The root mean square deviation (RMSD) between backbone atom coordinates was computed between identical and homologous proteins only. The RMSD analysis focused on the maximal values obtained from the all-against-all comparison of proteins binding the same fragment.

The conservation of the conformation of the same ligand in all its bound forms was evaluated by rigid body fit using PyMOL (v2.3.5, Schrodinger, LLC).

Structural similarity to XChem’s functionally diverse fragments and SpotXplorer library was evaluated using ECFP4 molecular fingerprints and Tanimoto’s coefficient (Tc) using Pipeline Pilot. The maximal similarity was reported for each versatile PDB fragment for the two compared sets.

Chemical scaffolds in the versatile PDB fragments were identified following the Bemis and Murcko approach (Bemis and Murcko, 1996) implemented in ADMET predictor (ADMET Predictor, Simulations Plus, Inc., Lancaster, California, United States). Fragments were standardized, neutralized, then classified using the option “Frameworks”.

How the versatile PDB fragments populate the chemical space defined by the commercial fragments libraries was investigated by projecting the versatile PDB fragments in the published GTM map (Revillo Imbernon et al., 2022). Fragments were standardized using Standardizer (v16.10.17.0; ChemAxon Ltd.) and transformed into IIAB (2–4)_cycle ISIDA descriptors using ISIDA/Fragmentor (v.2019, Faculté de Chimie, Université de Strasbourg, France). The map was explored using GTM tools (Kireeva et al., 2012).

The versatile PDB fragments were searched in the drug-like collection of on-the-shelf commercially available compounds (Perebyinis and Rognan, 2022) and in Drugbank (Wishart et al., 2018). Compound standardization and substructure search were performed using RDKit (RDKit: Open-source cheminformatics, https://www.rdkit.org, https://doi.org/10.5281/zenodo.7415128). The molecules were neutralized, and their canonical tautomeric state was determined. The substructure search was performed using the fragment as the substructure query, and the commercial library or DrugBank compounds as the target. The chirality of both the query and the target were considered during the search. The contribution of each fragment to its corresponding superstructure was determined as the fraction of bonds in the query, over the number of bonds in the target. The result of the search was considered an exact match if the ratio between the number of bonds in the query and the target was equal to 1, otherwise it was considered as a substructure.

Datasets are available on Zenodo (doi: 10.5281/zenodo.7554649). The repository contains the MOL2 files of the crystallographic structures of the PDB versatile fragments, their binding proteins, their binding cavity, and interaction pseudoatoms. The repository also contains five CSV files: One including the versatile PDB fragments, their SMILES string, their number of binding modes and their number of binding cavities; another one containing the HET code and SMILES of the 521 fragments maintained after the PDB and ligand type filtering; the third CSV file groups the starting PDB information downloaded from the website (RCSB, 2022); another one containing the RSCC scores of fragments and binding sites; the last one summarizes the results of the search for the versatile PDB fragments in Drugbank and commercial drug-like libraries.

## 3 Results

**FIGURE 1:**
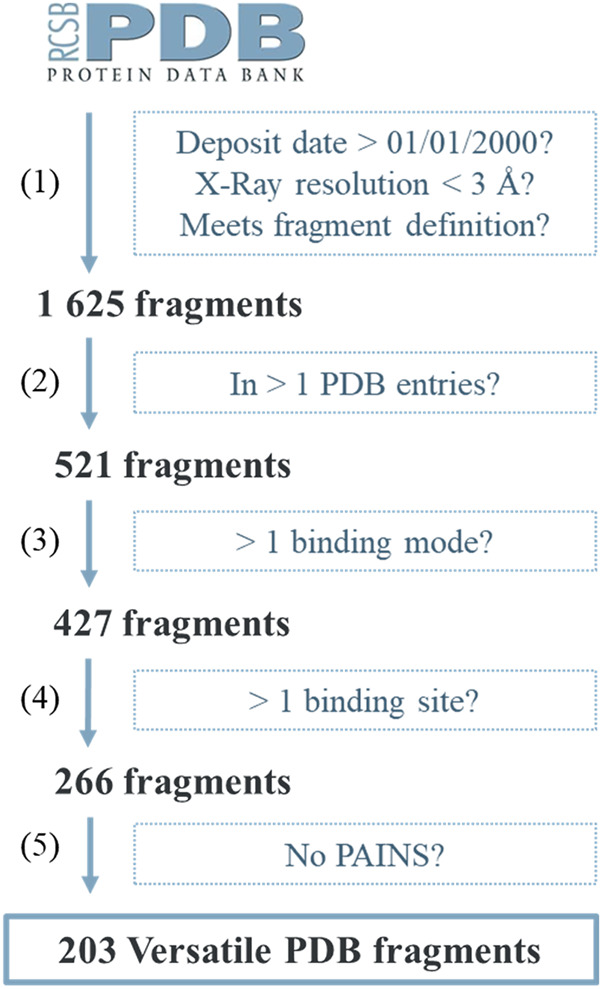
Overall workflow.

The versatile fragments were selected from the PDB following a protocol comprising five filtering steps: 1) Unwanted molecules that do not meet the definition of a fragment, 2) molecules present in a single entry, 3) molecules that show only a single binding mode, 4) molecules that are described only in binding sites that are similar, and 5) PAINS compounds which are likely to interfere with bioassays (Figure 1). At the end of step 2, a total of 521 fragments were retained. For each of them, binding modes were grouped following a density-based clustering using pairwise comparisons by subgraph matching. The distance threshold for clustering was defined on the inflection point in a polynomial projection of the distribution of the comparison data of all the 521 fragments. The visual examination of the clusters revealed cases of separation of binding modes when one is included in the other. Therefore, we applied a correction aiming to group them together. A total of 427 fragments showed two or more binding modes. The ability of these fragments to adapt to different protein environments was then assessed using a new protein comparison method which focuses on local 3D alignment. Protein cavities were delineated based on the shape and size of the bound fragment. The distribution of similarity scores is bimodal, with a first maximum at 0.86 corresponding to the median value obtained for the comparison of two copies of the same site in the same protein, and a second maximum at 0.34 corresponding to the median value for the random comparison of two unrelated sites. Density-based clustering using the recommended threshold for detecting similar sites (0.47) tended to cluster overlapping sub-sites of a pocket while well separating cavities with no visible commonalities. A total of 266 fragments were found in complex with two or more dissimilar binding sites. Of this set, 63 fragments were discarded because they contained quaternary pyridines, phosphorus, catechol among other structural motifs prevalent in problematic screening compounds.

The structure quality was assessed based on the correlated quality indicator RSCC, available for 4,997 of the 5,220 structures of the 203 versatile fragments. The protein binding site structures are mostly very well resolved (97% of the binding site structures fit to the density). Overall, the fragment structures are also well defined (72% and 18% of the fragment structures fit and partly fit the electron density, respectively). The 10% of structures where part of the fragment is not in the density concern 115 fragments. All data was used in the analysis that follows. If the lower quality structures had been discarded, one fragment would have been excluded for lack of binding mode diversity (HET code: NBO) and eleven fragments would have been excluded for lack of binding site diversity (HET code: IBP, M2S, PEA, 2EC, LTN, 4BX, 2UP, 1FL, MOK, FMK, and AJD).

**FIGURE 2:**
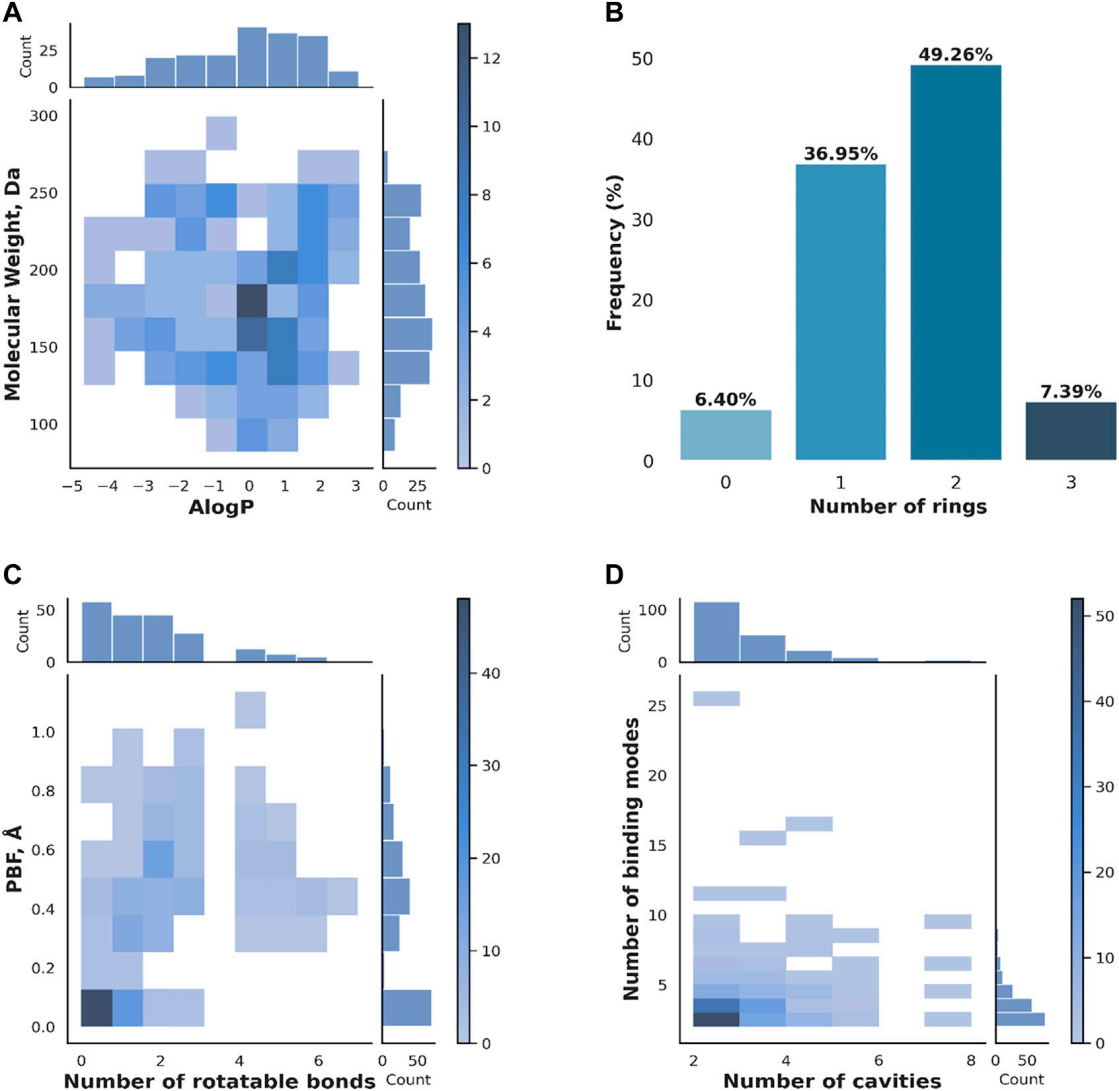
Properties of the versatile PDB fragments. **(A)** Molecular weight vs*.* AlogP **(B)** Number of rings **(C)** PBF vs*.* Number of rotatable bonds **(D)** Number of binding modes vs*.* Number of binding cavities. The distributions of individual properties are given on the top and side of the heat maps in **(A)**, **(C)** and **(D)**. The color scale on the side of the heat maps in **(A)**, **(C)** and **(D)** refers to the number of fragments.

The 203 versatile fragments identified in the PDB mostly comply with the rule of three, with 130 presenting no rules’ violation, 46 one rules’ violation, 26 two rules’ violations, and only one three rules’ violations. Rules violations are mostly linked to the number of hydrogen bond donor (24) and acceptor groups (49), the number of rotatable bonds (27), and only in one instance the logP. Figure 2 shows the absence of molecular obesity in the set (panel A), and provides an overview of the molecular complexity (panel B) and three-dimensionality (panel C) of the selected fragments. For more details, the molecular weight of 91 fragments falls in the 175–250 range. All fragments contain 6 to 18 non-hydrogen atoms (Figure 2A). Fragments are weakly lipophilic (107 fragments with 0 ≤ logP <3) or hydrophilic (95 fragments with logP <0). A total of 175 fragments contains one or two rings (Figure 2B). Consistent with ring aromaticity, there are 66 flat fragments, as indicated by the zero PBF value (Figure 2C), yet they only represent one-third of the entire set. The number of rotatable bonds does not exceed three for almost 87% of the fragments.

The 203 versatile fragments are present in 2,303 crystallographic PDB structures, representing 5,220 structures of fragment-protein complexes. About 82% of versatile PDB fragments bind to two or three different cavities, and accordingly using a small number of binding modes (Figure 2D). Exceptions are nucleosides such as thymidine, which shows 26 binding modes in two cavities, and flexible linear fragments such as the amino acid arginine, which shows six binding modes in two cavities. A total of twelve fragments are present in five or more cavities: six amino acids (lysine, canavanin, methionine, phenylalanine, leucine and pidolic acid), one nucleobase (guanine), two molecules naturally present in the organism and three exogenous molecules. The highly versatile natural molecules are both small ring system substituted with oxygenated group: the gamma-butyrolactone, which is a precursor of gamma-hydroxybutyrate (HET code: GBL) and the vitamin C or ascorbic acid (HET code: ASC). The three exogenous molecules target five to seven cavities: Afegostat, an iminosugar which failed a phase II clinical trial for Gaucher’s Disease (PDB code: IFM), 4-bromo-1H-pyrazole (HET code: BYZ), and thiophene-2-carboxylic acid (HET code: C21).

Sequence comparison between the proteins binding the same fragment revealed that 140 fragments bind to non-related proteins (identity <25%), specifically with two to three proteins for 93 fragments and more than 40 proteins for four fragments (resorcinol RCO: 82, styrene oxide RSO: 58, uridine URI: 41, and phenylalanine PHE: 64). Only eight of the fragments are in complex with the same protein or close homologs (identity ≥90%), whose overall structure is well conserved (maximum pairwise RMSD ≤1.42 Å). The remaining 55 fragments bind to more distant homologs (25% ≤ identity ≤90%), whose overall structure is either well conserved (maximal pairwise RMSD ∼1 Å for 14 fragments) or else very different (maximal pairwise RMSD ∼6 Å for 41 fragments). Thus, a total of 22 of the fragments explore several local sites in proteins that are similar in sequence and overall structure.

The conformations of the fragments were compared to assess whether structural plasticity can account for the versatility of binding. About 43% of the 203 versatile fragments show a conformation which is conserved in all their binding sites (maximum pairwise RMSD ≤0.5 Å) and only 11 fragments use very different conformations to adapt to their different protein environments (maximum pairwise RMSD >2 Å). On average, fragment conformation is more variable for fragments that bind to homologs than for fragments that bind to proteins that are unrelated in sequence: the mean value of the maximum pairwise RMSD is equal to 0.78 Å ± 0.69 for the 140 fragments binding to non-related proteins, to 0.88 Å ± 0.72 for 55 fragment binding to distant homologs, and 0.97 Å ± 0.58 for the eight fragments binding to the same protein or close homologs.

**FIGURE 3:**
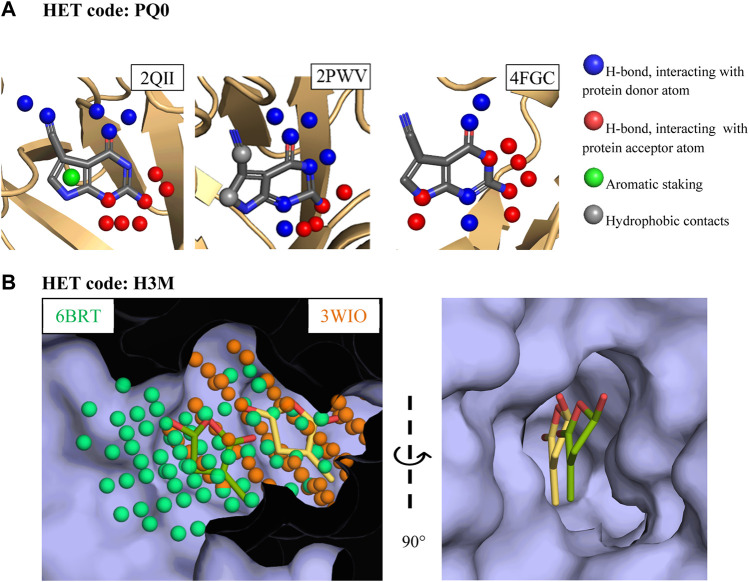
Examples of three-dimensional structures of PDB versatile fragments **(A)** Interactions formed by pyrrolo-pyrimidine in tRNA-guanine transglycolase and nitryl reductase QueF. **(B)** Substituted furanone binding to two adjacent subpockets in strigolactone esterase D14. The cavity points representing the two subpockets and used for the comparison of the two sites are shown in the left image.

Two examples of versatile fragments are discussed below and illustrate how a fragment adapts its binding mode to unrelated proteins (Figure 3A), and to different sites in the same protein (Figure 3B). The first example involves a pyrrolo-pyrimidine fragment (HET code: PQ0). This fragment shows three binding modes in two protein cavities found in two types of proteins, tRNA-guanine transglycolase (PDB codes: 1IT8, 1P0B, 2PWV, and 2QII) and nitryl reductase QueF (PDB code: 4FGC). The three binding modes are dominated by hydrogen bonds. Changes involve the pyrrole moiety, which does not interact with the protein or interact with a tyrosine side chain depending on local structural variation of tRNA-guanine transglycolases binding site. More importantly, different protonation states of the fragment allow for different hydrogen bonding patterns in the two proteins. Last, although it is not the focus of the present study, this fragment also binds to RNA (PDB code: 3GCA) (Berman et al., 2000).

The second example consists of a furanone (HET code: H3M), which is in complex twice with the strigolactone esterase D14 (PBD code: 6BRT and 3WIO), yet shows a 4 Å shift in the main cavity, thereby defining two non-overlapping subpockets (Figure 3B). The two corresponding binding modes are dominated by *π* interactions which are frequently observed in RNA-protein complexes (Wilson et al., 2021), but they differ in the hydrogen bonds formed with the protein, which either involve a tryptophan indole ring, or a cysteine sulfhydryl and a protein-bound water molecule. Of note, the binding mode comparison method used here ignored weak hydrogen bonds and water mediated interactions yet was nevertheless able to differentiate the two binding modes. Interestingly, the three-dimensional structures of furanone ring are not identical in the two complexes, suggesting that despite the absence of rotatable bonds this scaffold is able to adapt its shape to the local environment.

The versatile PDB fragments were compared to two academic collections, the XChem’s functionally diverse fragments and SpotXplorer library. The distribution of maximal similarity between versatile PDB fragments and XChem’s functionally diverse fragments had a median maximal value equal to 0.21 (Supplementary Figure S1). Thirteen versatile PBD fragments share common chemical structures with XChem’s functionally diverse fragments (Tc > 0.45). One single fragment is common to both sets. This fragment is a sulfapyridine (HET code: SFY), which shows two binding modes in three cavities. It contains multiple pharmacophoric features (two hydrophobic rings, three hydrogen bond donors, two hydrogen bond acceptors), allowing adaptation to different protein environments. The chemical structures of versatile PDB fragments and SpotXplorer library do not overlap, with a median maximal similarity value equal to 0.23. A total of six versatile PBD fragments share common chemical structures with SpotXplorer library (Tc > 0.45).

**FIGURE 4:**
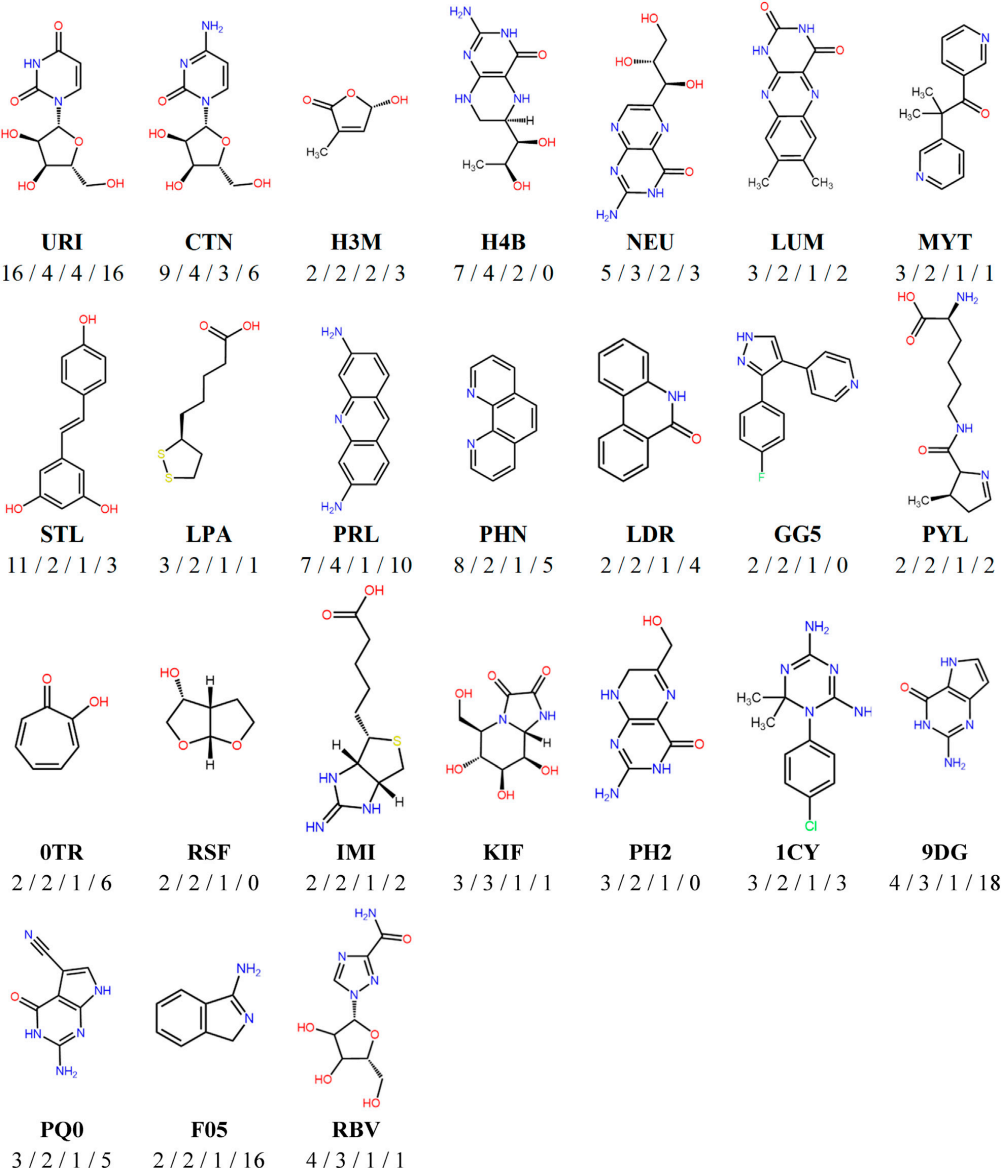
Representative chemical structures of the versatile PDB fragments which are absent or poorly represented in commercial fragments libraries. Below each fragment structure are indicated the HET code, then the number of binding modes/the number of different cavities/the number of versatile PDB fragments containing the same scaffold/the number of commercial fragments containing the same scaffold.

**FIGURE 5:**
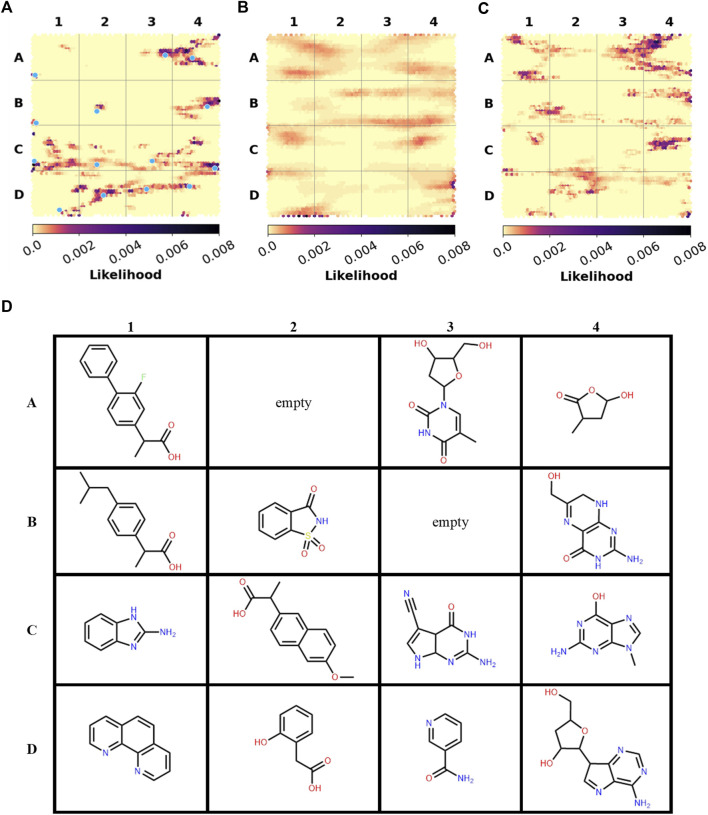
Spatial distribution of the fragments in GTM Landscapes **(A)** Versatile PDB fragments **(B)** All commercially-available fragments **(C)** Ensemble of nature-product like commercial libraries. **(D)** Examples of versatile PDB fragments. Their projection points are marked with blue dots on the map **(A)**. Figures 5B, C are adapted from (Revillo Imbernon et al., 2022).

The versatile PDB fragments were compared to the commercially available fragments using two approaches: shared Bemis and Murcko scaffolds and generative topographic map (GTM), which represents the chemical space in a 2D map, here based on topological descriptors (Kireeva et al., 2012). The 203 fragments are covered by 90 scaffolds. The commercial chemical space of fragments was described with about 60,000 scaffolds (https://gtmfrag.drugdesign.unistra.fr/) ranging from very simple (benzene) to very complex (adamantane) (Revillo Imbernon et al., 2022). We identified four absent scaffolds and 20 rare scaffolds, which were observed in twenty or less commercially available fragments (Figure 4). These 24 scaffolds represent a total of 32 fragments (Supplementary Figure S2). They show on average more oxygen atoms than commercial fragments (1–6 O in 79% of PDB scaffolds; 1–3 O in 31% of commercial scaffolds), and conversely fewer nitrogen atoms (1–5 N in 79% of PDB scaffolds; 1–6 N in 93% of commercial scaffolds). The coverage of the fragment space by the versatile PDB fragments was further analyzed through a GTM model recently developed to compare commercial libraries, based on the likelihood of the compounds projected onto the common frame (Figure 5). The versatile PDB fragments populate three regions which show a low density among the general commercial fragments set (Figure 5B): **A4**, **C2**, and **C4**. The region **A4** groups together three-dimensional molecules, mostly non-aromatic heterocycles which contain oxygen atoms. The region **C2** includes aromatic rings fused and substituted with alcohol, ketone or carboxylic acid groups. The region **C4** shows many purine derivatives with amino substituents on the pyrimidine ring. Noteworthy, the patterns observed in regions **A4** and **C2** have a low likelihood among the general commercial fragments set (Figure 5B) but are characteristic of nature-product like fragments of commercial libraries (Figure 5C).

The 203 versatile PDB fragments were further characterized by querying a repository of approved drugs, Drugbank (Wishart et al., 2018), and screening collections of commercial catalogs. A total of 45 fragments are registered as approved small molecule drugs in DrugBank. Among these, seven are also nutraceuticals and sixteen in clinical trials. Around half of the fragments (98) were identified as substructures of 1,436 approved drugs. The fragments generally represent a small portion of the corresponding superstructures, with the bonds in the fragment representing less than 50% of the bonds in the superstructure for 88% of the fragment-superstructure pairs. While 22 fragments are substructures of only a single drug, 48 are found in at least five drugs. Larger fragments (MW > 175) account for 28 of the 45 identified drugs, while 37 represent a substructure of 134 drugs. The exploration of commercial catalogs identified 95 purchasable fragments, and 163 fragments substructures of a huge number of commercially available screening compounds (4,769,862). The fragments represent however less than 50% of the superstructures’ bonds in 97% of the fragment-compound pairs.

## 4 Discussion

The PDB is an essential resource for structure-based drug discovery. Most drugs that have been approved by the FDA over the past decade were developed when structural information was freely available for the protein target (Westbrook and Burley, 2019). Additionally, with 38,790 entries in the Chemical Component Dictionary in 2022, the PDB provides important material for characterizing recognition between small molecular entities and proteins. The number of fragment-sized chemical entities (MW ≤ 300 Da) amounts to 13,965. Our analysis of the PDB identified only 203 versatile fragments, able to adopt at least two binding modes and to bind to at least two distinct sites. Most of these fragments target multiple proteins that differ in sequence, but we also highlighted eight fragments that bind differently to the same protein or close homologs.

Do the properties of the 203 versatile PDB fragments meet FBDD expectations? Lipophilicity and molecular weight are two widely used criteria, as they are related to solubility, metabolism and selectivity, and a *sweet spot* has been suggested around a molecular weight 400 Da and a logP around 3 (Hann, 2011; Hann and Keserü, 2012). These values are shifted down in commercial fragment libraries with molecular weight in the range 200–300 Da and logP in the range 0–2 (Revillo Imbernon et al., 2022). A minimal molecular weight around 175 Da was suggested by the study of the conservation of the binding mode of the fragments in their drug-like superstructures (Jacquemard and Kellenberger, 2019). The same threshold was proposed following the analysis of XChem’s screening campaigns which revealed that fragments that have never been shown to bind to a target tend to have a low molecular weight compared to fragments that bind to one or more targets (Carbery et al., 2022). According to the size criterion, about half of the versatile PDB fragments may provide a valid base for fragment growing (105 fragments with MW > 175 Da). A total of 38 fragments show a logP in the 0–2 range, yet there are also 50 hydrophilic fragments (logP <0). The combinations of lipophilicity and molecular weight which are little explored by recently developed synthetic oral drugs should not be overlooked since they may correspond to molecules of therapeutic interest such as natural products (e.g., antibiotics) (Young et al., 2022). Thus, we did not exclude hydrophilic fragments from the analysis. In addition, these fragments have the advantage of good aqueous solubility.

Are the 203 versatile PDB fragments original? To answer this question, we compared this set to XChem’s functionally diverse fragments and SpotXplorer library, to the chemical space covered by commercial fragment libraries, and to screening collections of drug-like molecules. The XChem’s functionally diverse fragments and SpotXplorer library are two collections comprising a wide diversity of pharmacophores evidenced by the binding modes observed in the structural data (Bajusz et al., 2021; Carbery et al., 2022). The comparison of chemical structures revealed that these two collections and the 203 versatile PDB fragments hardly overlap. The XChem’s functionally diverse fragments and SpotXplorer library being assembled from commercial catalogs, we therefore sought to assess whether the scaffolds of the 203 versatile PDB fragments were indeed present in the fragment’s libraries provided by chemical suppliers. According to our 2021 inventory (Revillo Imbernon et al., 2022), 198 versatile PDB fragments or structural analogs are commercially available, but 27 of them are rare in commercial fragment libraries. The five fragments whose scaffold is missing from commercial fragment libraries also cannot be purchased from commercial drug-like collections. These fragments are all the more original as only two of them are substructures of an available compound (HET code GG5 and RSF, representing 71% and 47% of the superstructure bonds in their respective best fragment/compound pair). In summary, if a large part of the versatile PDB fragments can be purchased from chemical suppliers, 24 of their scaffolds are little or not represented in commercial fragment libraries (Figure 4).

Many of the versatile PDB fragments that are not or rarely present in commercial fragment libraries are enriched in oxygen atom. Oxygen containing functional groups, such as ether, are more common in natural products than in synthetic molecules (Ertl and Schuhmann, 2019). Among oxygen-containing versatile PDB fragments are eight nucleosides and close analogs. The phosphorylated nucleoside, i.e., nucleotides, are common cofactors, therefore recognized by numerous protein sites. They have already widely inspired medicinal chemists, allowing for example the development of many anti-viral drugs (Seley-Radtke and Yates, 2018; Yates and Seley-Radtke, 2019).

Have all the 203 versatile PDB fragments already been exploited in drug discovery? To answer this question, we explored Drugbank thus identifying 45 approved drugs. According to Drugbank annotations, 29 of these drugs have more than one known target. For example, acetazolamide used to treat edema, certain types of epilepsy and glaucoma, has been reported to target nine types of carbonic anhydrase and aquaporin-1. The PDB structures provide eight binding modes of acetazolamide in multiple enzyme types: carbonic anhydrase, chitinase, deacetylase and synthase (HET code: AZM). The 16 other drugs have a single known target or no targets. For example, sulfapyridine is a sulfonamide antibiotic targeting dihydropteroate synthase type-1. The PDB structures provide four binding modes of sulfapyridine in multiple protein types: reductase, kinase, proteinase, erythrocine membrane protein and synthase (HET code SFY). As an example of drug with no known target, piracetam was crystallized in glutamate receptor 2 and glutamate receptor 3 (HET code PZI). Interestingly, 123 of the versatile PDB fragments are substructures of approved drugs, including drugs with a single known target. The data revealed examples of promiscuous fragment embedded into a selective drug, such as indole-3-carboxylic acid (HET code ICO) which was crystallized in complex with four types of protein (androgen receptor, integrase, malate synthase G, and a transcriptional regulator), and which also represents about half of tropisetron, a 5HT-3 receptor antagonist used as an antiemetic in the treatment of chemotherapy-induced nausea and vomiting (Ki = 2.70 nM, BindingDB entry BDBM50108392 (Chen et al., 2001)). In summary, we have identified 80 versatile PDB fragments that have not been yet exploited in drugs. Moreover, structural data on the 203 versatile PDB fragments reveal new off-targets for existing drugs and show that a versatile fragment can evolve into a drug with high affinity for its target. This data, freely available on zenodo, provides fragment-focused insights complementing previous studies of multi-target PDB ligands (Sturm et al., 2012; Barelier et al., 2015; Pottel et al., 2018; Feldmann and Bajorath, 2020; Pinzi and Rastelli, 2020) that can be used to better understand polypharmacology (Chaudhari et al., 2017; Proschak et al., 2019).

## 5 Conclusion

The clustering of the binding modes and binding sites of PDB fragments issued a set of 203 low molecular weight compounds able to adapt to different protein environments and to exploit several combinations of interacting atoms. The versatile PDB fragments constitute an ensemble which contains many structures that can be purchased directly yet also a few scaffolds that are poorly represented in fragment screening collections. The presence of oxygenated compounds in rare and original scaffolds calls for renewed interest in natural products. Since the PDB provides structural insights into well-studied biological functional systems, a significant amount of versatile PDB fragments (45/203) are approved drugs. Moreover, many promiscuous fragments are a substructure of selective drug, suggesting their utility for FBDD.

## Data Availability

The datasets presented in this study can be found in online repositories. The names of the repository/repositories and accession number(s) can be found in the article/Supplementary Material.
