# Supplementary material for: Mining the Protein Data Bank to inspire fragment library design
Source: Front Chem. 2023 Feb 10;11:1089714. doi: 10.3389/fchem.2023.1089714 (PMC9950109; doi:10.3389/fchem.2023.1089714)
Supplement: Supplementary file 1 [file DataSheet1.PDF]

Mining the Protein Data Bank to inspire fragment library design

Julia Revillo Imbernon[1], Luca Chiesa[1], Esther Kellenberger[1]

[1] Laboratoire d'Innovation Thérapeutique, UMR7200 CNRS Université de Strasbourg, Faculté de Pharmacie, Illkirch-Graffenstaden, France

Table 1: Visually discarded fragments by category.

**Discarded group**	**Discarded HET**
Aliphatic	DCX
Ammonium	144, BTM, TBA, 211, DCD, N6C
Monosaccharides	IPT, G4D, ISD, IPD, KDG, X1P, ASO, LGC, M7P, GAR, GDL, GLR, GPM, SOE, 1KM, NHF, R2B, M7B, LIP, 149
Phosphate	PPF, 3PP, PEP, 13P, 2PG, DPF, DPJ, P3S, G88, SUF, AS9, FOM, PGH, OPE, CP, PPR, EIP, IPR, IPE, 210, PC, KPC, PCT, S0H, UVW, P23, P25, DST, MPJ, FPE, DED, CDI, PLU, PAE, MDN, TCE, ISY, 2HE, M44, ODV, TB6
Polyhalogenated	PCI, HLT, ICF, PFB, CFH, T6C
Sulfate	ESA, TAU, COM, SPV, PS9
Too small	TBU, DMF, PTL, BTL, AKR, MOE, PTD, TBF, BMD, EEE, NMU, OXM
Two in site	ID3, 2MY

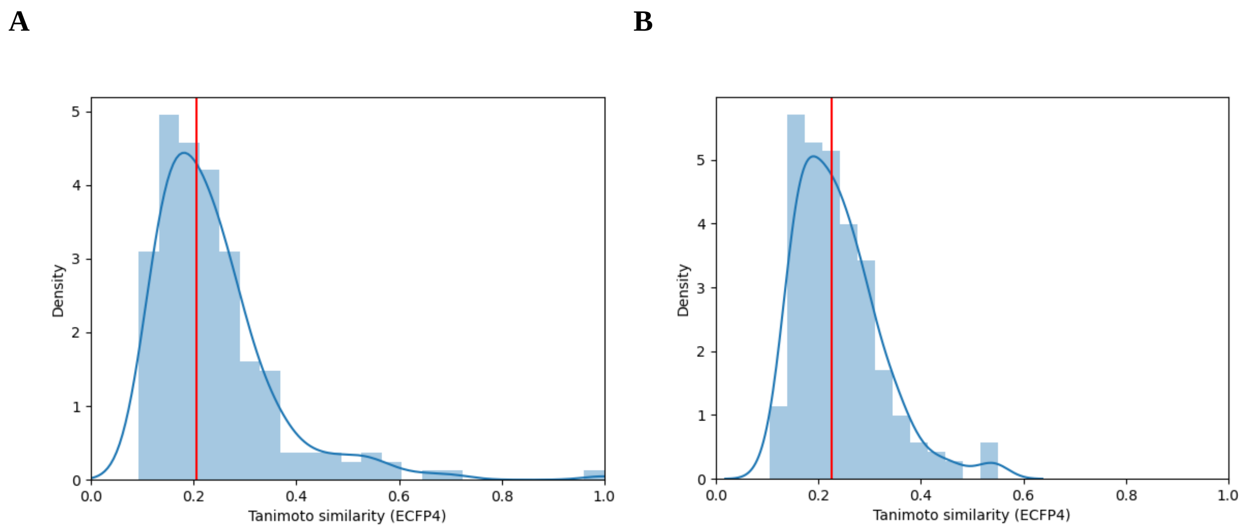

Figure 1: Distribution of Tanimoto Similarity between the versatile PDB fragments and (A) XChem top 100 fragment selection (B) SpotXplorer. The red line represents the median maximal value.

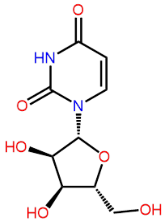

URI

16/ 4 / 4 / 16

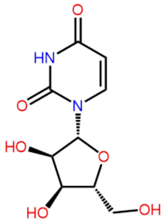

5UD

5 / 2 / 4 / 16

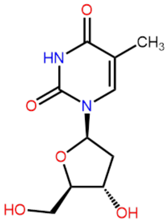

THM

26/ 2 / 4 / 16

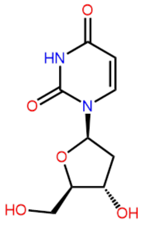

DUR

15/ 3 / 4 / 16

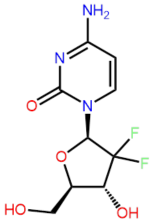

GEO

4 / 4 / 3 / 6

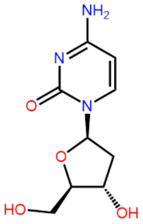

DCZ

6 / 2 / 3 / 6

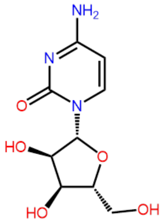

CTN

9 / 4 / 3 / 6

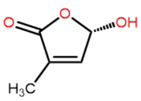

H3M

2 / 2 / 2 / 3

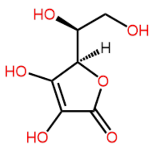

ASC

8 / 5 / 2 / 3

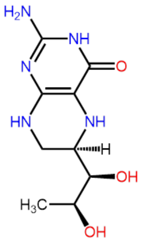

H4B

7 / 4 / 2 / 0

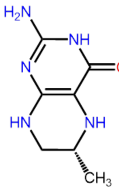

B62

3 / 2 / 2 / 0

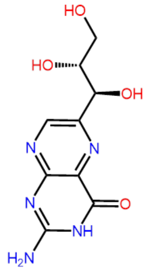

NEU

5 / 3 / 2 / 3

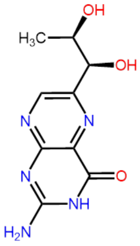

BIO

6 / 2 / 2 / 3

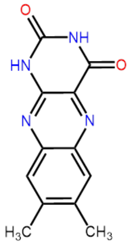

LUM

3 / 2 / 1 / 2

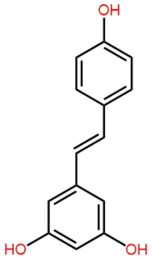

STL

11 / 2 / 1 / 3

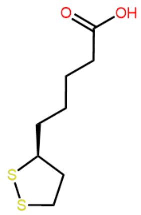

LPA

3 / 2 / 1 / 1

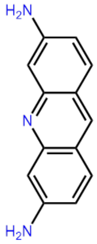

PRL

7 / 4 / 1 / 10

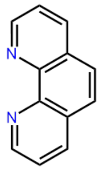

PHN

8 / 2 / 1 / 5

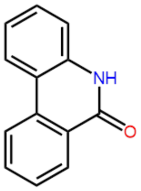

LDR

2 / 2 / 1 / 4

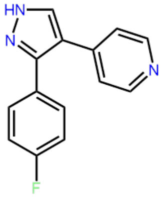

GG5

2 / 2 / 1 / 0

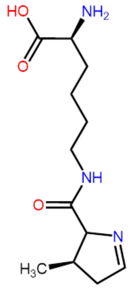

PYL

2 / 2 / 1 / 2

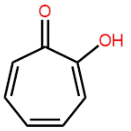

0TR

2 / 2 / 1 / 6

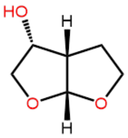

RSF

2 / 2 / 1 / 0

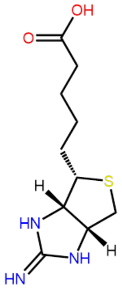

IMI

2 / 2 / 1 / 2

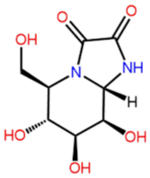

KIF

3 / 3 / 1 / 1

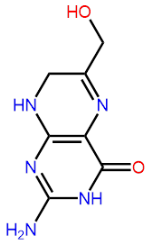

PH2

3 / 2 / 1 / 0

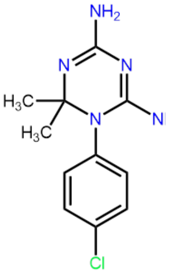

1CY

3 / 2 / 1 / 3

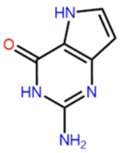

9DG

4 / 3 / 1 / 18

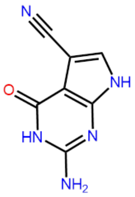

PQ0

3 / 2 / 1 / 5

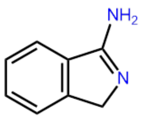

F05

2 / 2 / 1 / 16

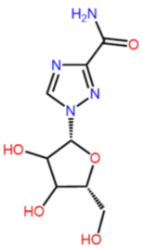

RBV

4 / 3 / 1 / 1

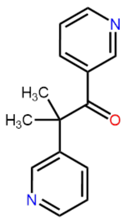

MYT

3 / 2 / 1 / 1

Figure 2: Representative chemical structure of versatile and original PDB fragments. Below each fragment structure are indicated the HET code, then the number of binding modes / the number of different cavities / the number of versatile PDB fragments containing the same scaffold / the number of commercial fragments containing the same scaffold.
